# 

**Published:** 1881-07

**Authors:** 


### CONTENTS:

| | PAGE. |
|---|---|
| ORIGINAL COMMUNICATIONS: | |
| Notes on Puerperal cases. By Thomas Lothrop, M. D., | 529 |
| Arrow Wounds. By H. S. Kilbourne, M. D., | 538 |
| CLINICAL REPORTS: | |
| Surgical Cases in Private Practice of Dr. C. C. F. Gay, | 545 |
| Incision of the Tunica Vaginalis and Albuginea Testes for Relief of Testitis Occurring as a Sequel of Parotitis. | 548 |
| A Case of Double Ovariotomy.—Operation by Professor James P. White. Reported by C. M. Daniels, M. D., | 549 |
| TRANSLATIONS: | |
| Iodoform as a Dressing, Especially in Tubercular Ulcerations of Bones and Joints.—By Mikulicz (Vienna.) From the German by Frederick Peterson, M. D., | 552 |
| SELECTIONS: | |
| On Tenia and Teniafuges. By Dr. Carl Bettleheim, | 554 |
| One Phase of the Germ Theory. By E. F. Brush, M. D., | 557 |
| SOCIETY REPORTS: | |
| Semi-Annual meeting of the Erie County Medical Society, | 562 |
| EDITORIAL: | |
| New Work for the State Board of Health, | 565 |
| Warner's Sugar-Coated Pills, | 566 |
| Lactopeptine, | 567 |
| Surgical Instruments. | 567 |
| REVIEWS, | 567 |

